# Decannulation: in the ICU or in the ward? Does it really matter?

**DOI:** 10.1186/cc9588

**Published:** 2011-03-11

**Authors:** O Milercy, J López, J Figueira, J Manzanares, M Hernández

**Affiliations:** 1 La Paz Hospital, Madrid, Spain

## Introduction

The aim of our study was to evaluate the in-hospital mortality of patients who underwent tracheostomy during their ICU admission, and were discharged to different areas of the hospital prior to decannulation.

## Methods

A prospective observational study of a group of patients who underwent tracheostomy in our ICU from January 2001 to December 2007 and were discharged to different areas of the hospital prior to decannulation. The mortality of patients decannulated or not in the wards was reviewed.

## Results

Between January 2001 and December 2007, 6,333 patients were admitted to our unit. A total of 1,528 needed mechanical ventilation (MV) for more than 48 hours. Four hundred and forty-three underwent tracheostomy (29% of patients needed prolonged MV). Mean age was 56 years, 66% were male. Mean APACHE II score was 20. The main diagnoses were polytrauma that included head injury (24.2%), other structural neurological diseases (21%), prolonged weaning of several etiologies - sepsis, post-surgical (35%). Tracheostomy was performed with the percutaneous dilatational technique (PDT) in most cases (90%). The most frequent complication was subglottic stenosis presenting in 15 patients. Ninety-two patients (20.77%) died in the ICU and 351 were discharged to different wards. Of these 351, 161 (45.8%) could be decannulated in the ICU and 109 (31%) in the wards. Eighty-one patients (23%) could not be decannulated. The ward mortality in patients decannulated in the ICU was 5.6% (9/161), for those decannulated in the wards was 10% (11/109). In patients not decannulated the mortality reached 37% (30/81). There were no differences of statistical significance in mortality between patients decannulated in the ICU and patients decannulated in the wards (5.6% vs. 10%; OR = 1.9 CI = 0.8 to 4.2). The main diagnoses in the patients who died on the wards were: 31 residual encephalopathy (post-anoxic, post-traumatic, others), five severe chronic respiratory failure, three spinal cord injury, two neuromuscular disease.

## Conclusions

Mortality was not related to whether decannulation was done in the ICU or on the ward. Although mortality was higher in the group of patients that could not be decannulated in either setting due to their poor neurological or functional status. Several authors suggest tracheostomy in these patients only delays their death without improving overall in-hospital survival due to their poor vital prognosis.
